# MRD conversion during oral azacitidine maintenance correlates with outcomes in predominantly favorable-risk AML

**DOI:** 10.1007/s00277-026-07054-5

**Published:** 2026-05-09

**Authors:** Noa Hurvitz, Irina Amitai, Ilana Levy Yurkovski, Ariella Tvito, Yishai Ofran, Tzvika Porges, Ofir Wolach, Adi Sherban, Noa Gross Even-Zohar, Vladimir Vainstein, Arnon Haran, Shlomzion Aumann, Robert Mikhelashvili, Avraham Frisch, Boaz Nachmias

**Affiliations:** 1 Leukemia Service, Department of Hematology, Hadassah Medical Center and, Faculty of Medicine, Hebrew University of Jerusalem, Jerusalem, Israel; 2 Hematology Division, Chaim Sheba Medical Center, Gray Faculty of Medical & Health Sciences, Faculty of Medicine, Tel Aviv University, Tel Aviv, Israel; 3 Hematology Division, Bnai Zion Medical Canter, Haifa, Israel; 4 The Ruth and Bruce Rappaport Faculty of Medicine, Technion, Israel Institute of Technology, Haifa, Israel; 5 Hematology and Stem Cell Transplantation Department and the Eisenberg RD Authority, Shaare Zedek Medical Center, Hebrew University Jerusalem, Jerusalem, Israel; 6 Institute of Hematology, Soroka University Medical Center, Beer Sheva, Israel; 7 Institute of Hematology, Davidoff Cancer Center, Rabin Medical Center, Gray Faculty of Medical & Health Sciences, Faculty of Medicine, Tel Aviv University, Petah-Tikva, Israel; 8 Department of Hematology and Bone Marrow Transplantation, Rambam Health Care Campus, Haifa, Israel

**Keywords:** Acute myeloid leukemia, Maintenance, Oral azacitidine, Measurable residual disease, Onureg

## Abstract

**Background:**

Oral azacitidine improves survival in patients with AML in first complete remission who are not candidates for allogeneic transplantation. Most favorable-risk AML patients harbor molecular markers enabling sensitive measurable residual disease (MRD) monitoring. While MRD is a well-established prognostic factor in AML, the clinical significance of MRD dynamics during maintenance therapy remains incompletely defined.

**Methods:**

We conducted a multicenter, real-world retrospective study of 30 patients with AML, predominantly ELN favorable-risk, treated with oral azacitidine maintenance following intensive induction-consolidation. MRD was assessed longitudinally using standardized molecular and flow-based assays. Outcomes were analyzed according to MRD status at maintenance initiation and subsequent MRD conversion.

**Results:**

At initiation of oral azacitidine, 47% of patients were MRD-positive. MRD conversion to negativity occurred in 64% of these patients during maintenance. Relapse-free survival (RFS) was comparable between patients who were MRD-negative at baseline and those who converted to MRD negativity (24-months restricted median RFS 577 vs. 638 days), whereas patients with persistent MRD positivity experienced early relapse (24-months restricted median RFS 63 days). Despite inferior RFS, overall survival did not significantly differ between MRD-defined groups. Patients relapsing after oral azacitidine retained sensitivity to subsequent therapies, including venetoclax–azacitidine–based regimens, with high rates of molecular response.

**Conclusions:**

Oral azacitidine was associated with high rates of MRD conversion, conferring RFS comparable to patients who were MRD-negative at treatment initiation. Persistent MRD identified patients at high-risk for early relapse, while effective salvage therapies mitigated overall survival differences. These findings support prospective evaluation of MRD-guided maintenance strategies in favorable-risk AML but should be considered hypothesis-generating given the retrospective design and limited cohort size. The trial is on behalf of the Israel Acute Leukemia Group.

## Introduction

Acute myeloid leukemia (AML) is a biologically and clinically heterogeneous malignancy characterized by high relapse rates and poor long-term survival, even among patients who achieve morphologic complete remission (CR) following intensive chemotherapy [1, 2]. Although allogeneic hematopoietic stem cell transplantation (HSCT) offers the most durable remission, many patients are ineligible due to age, comorbidities, or lack of a suitable donor. Studies of maintenance therapy in these patients were aimed to mitigate relapse and often failed to show a clear benefit.

Oral azacitidine (Onureg) is an oral formulation of the hypomethylating agent azacitidine designed to provide extended epigenetic therapy beyond induction and consolidation. In the pivotal phase III QUAZAR AML-001 trial, oral azacitidine significantly prolonged both overall survival (OS) and relapse-free survival (RFS) compared with placebo among patients in first CR/CRi who were not candidates for HSCT [3]. Importantly, oral azacitidine improved OS in MRD positive patients and was shown to increase the rate of MRD conversion as well as the durability of MRD negativity during maintenance. Treatment was generally well tolerated and maintained health-related quality of life [3–5].

The QUAZAR AML-001 trial enrolled patients with intermediate- and high-risk AML and included selected biologically favorable subsets; however, patients with favorable-risk cytogenetics were not included, and ELN favorable-risk patients were under-represented overall. Importantly, biologically favorable subsets such as NPM1-mutated AML were included, although not as a predefined favorable-risk cohort. In contemporary practice, many patients meeting ELN favorable-risk criteria do not proceed to allogeneic transplantation in first remission, whereas most intermediate- and high-risk patients who tolerate intensive chemotherapy are typically directed to HSCT. Consequently, the applicability of QUAZAR findings to real-world post-remission decision-making in favorable-risk AML remains incompletely defined.

The increasing use of next-generation sequencing (NGS) panels at diagnosis and measurable residual disease (MRD) assessment allows us to identify a subset of ELN-favorable patients who nonetheless carry an elevated risk of relapse [6–8]. Common examples include NPM1-mutated AML with co-occurring DNMT3A mutations[9–12], age > 60 years [13, 14], NPM1 mutated AML achieving low positive MRD at the end of consolidation therapy, or negative MRD with co-mutation in FLT3-ITD [6, 8, 15]. Similarly, core-binding factor (CBF) AML patients with RAS or KIT mutations, or low-positive MRD after consolidation therapy have been shown to have worse prognosis [16, 17].

We hypothesize that these “high-risk favorable” patients might derive a meaningful benefit from oral azacitidine maintenance, particularly because, under current clinical practice, they typically do not proceed to allogeneic HSCT in first remission.

To address these evidence gaps, we conducted a multicenter, retrospective, real-world cohort study of patients with favorable ELN risk AML, treated with oral azacitidine maintenance. The study aimed to describe patient characteristics, treatment patterns, response and survival outcomes, and adverse events, thereby providing additional insight into the use of oral azacitidine among patients with favourable- and intermediate-risk AML.

## Methods

### Study population

This is a retrospective, multicenter cohort study conducted across seven academic medical centers in Israel (Chaim-Sheba Medical Center, Rambam Medical Center, Shaare Zedek Medical Center, Bnai Zion Medical center, Soroka Medical Center, Rabin Medical Center and Hadassah Medical Center).

The cohort was predominantly composed of ELN 2022 favorable-risk AML patients (90%), with a small subset of intermediate-risk patients included, reflecting real-world treatment patterns, who received maintenance therapy with oral azacitidine during the study period [2]. Data were collected from institutional electronic medical records.

Collected variables included demographics, ELN risk group, cytogenetics and molecular markers, treatment regimens, remission dates, MRD results, maintenance treatment duration, adverse events, and survival outcomes. Molecular MRD data was available for NPM1 mutation and core binding factor (CBF) cytogenetic abnormalities (INV16 and 8;21). MRD assessment was performed in accordance with current ELN guideline recommendations [7]. Molecular MRD for NPM1-mutated and CBF AML was assessed using standardized quantitative PCR assays with ABL1-based normalization, using commercially validated standards across participating laboratories. This approach enables comparability of MRD measurements both between patients and longitudinally within individual patients. MRD test positivity by qPCR is defined as a cycling threshold < 40 in ≥ 2 of 3 replicates, CR with molecular MRD detectable at low-level positivity defined as < 2% [7]. Negative MRD is defined was undetectable prior to oral azacitidine initiation. During follow-up qPCR MRD was assessed every 3 months when obtained from BM samples, or alternatively, every 4—6 weeks if obtained from PB. In cases where molecular MRD was not available, flow based MRD was performed as per the ELN consensus guidelines with an estimated sensitivity of 0.1%. Flow cytometry–based MRD assessment was performed according to institutional standards and ELN guidelines, acknowledging potential inter-laboratory variability.

### Outcomes

The primary endpoints were relapse‑free survival (RFS), defined as the interval from oral azacitidine initiation to either molecular or overt relapse or death from any cause, and overall survival (OS), defined as the interval from oral azacitidine initiation to death from any cause.

Molecular relapse was defined as either 1-log increase or converting from negative to positive MRD in two consecutive tests. or Secondary endpoints included MRD conversion as defined by the transition to MRD negativity during oral azacitidine maintenance, treatment duration, discontinuation patterns, and safety.

### Statistics

Categorical variables are reported as counts and percentages. Continuous variables are presented as median and range. RFS and OS were estimated using the Kaplan–Meier method with censoring at last available follow‑up. To account for low event rates and the potential violation of proportional hazards, restricted mean survival time (RMST) [18], defined as area under the survival curve, was used for exploratory between-group comparisons for both RFS and OS. Statistical analyses were performed using GraphPad Prism (version 10.6.1; GraphPad Software, San Diego, CA, USA) and R (version 4.5.1) using the survival and survRM2 packages.

### Ethics

This study protocol was reviewed and approved by each center institutional review board and Hadassah Medical Center internal review board approval number (HMO0700—21-). Informed consent was waived due to the retrospective design and de‑identified data extraction.

## Results

### Patient characteristics and frontline therapy

A total of 30 patients were included in the cohort. Baseline characteristics are summarized in Table 1. The median age at diagnosis was 54.5 years (range, 24–72), and 60% of patients were female. The majority of cases were de novo AML (96.7%) and exhibited a predominantly favorable European LeukemiaNet (ELN) risk profile (90%), driven by mutations in *NPM1* (76.7%), *CEBPA* (6.7%), t(8:21) (3.3%), and inv(16) (3.3%), with the remaining patients classified as intermediate-risk AML. *FLT3*-ITD and *FLT3*-TKD mutations were identified in 10% and 20% of patients, respectively. A normal karyotype was observed in 83% of cases.

**Table 1:** Baseline characteristics

Parameter	Subgroup	No. of patients (Percentage)
**Total patients**	-	30
**Sex**	Male	12 (40%)
	Female	18 (60%)
**Median age (range), years**	-	54.5 (24–72)
**Disease history**	De novo	29 (96.7%)
	Therapy-related	1 (3.3%)
**ELN risk**	Favourable	27 (90%)
	Intermediate	3 (10%)
**Genetic mutations**	NPM1	23 (76.7%)
	FLT3-ITD	3 (10%)
	FLT3-TKD	6 (20%)
	Additional myeloid panel mutations	20 (66.7%)
**Karyotype**	Normal	25 (83.3%)
	Abnormal	5 (16.7%)
**Induction regimen**	7 + 3	26 (86.7%)
	CPX-351	2 (6.7%)
	HIDAC	1 (3.3%)
**Induction additions**	FLT3 inhibitor	8 (26.7%)
	GO	11 (36.7%)
**Number of inductions**	1 induction	29 (96.7%)
	Salvage	1 (3.3%)
**Median time to remission (range), days**	-	28 (15–39)
**Consolidation regimen**	HIDAC	14 (46.7%)
	HIDAC + GO	7 (23.3%)
	IDAC	6 (20%)
	CPX-351	1 (3.3%)
	No consolidation	2 (6.7%)
**Number of consolidations**	1	1 (3.3%)
	2	3 (10%)
	3	19 (63.3%)
	4	5 (16.7%)
**MRD after cycle 2**	Negative	13 (43.3%)
	Positive	15 (50%)
	Unknown	2 (6.7%)
**Median follow-up (range), months**	-	26.7 (8.5–64.9)
Favorable risk with additional high risk features n = 21*	Age above 60 y NPM1/DNMT3A CBF with RAS mutation Detectable MRD	11/21 (52%) 6/21 (28%) 1/21 (5%) 14/21 (67%)

LEGEND: ELN = European LeukemiaNet; MRD = measurable residual disease; CBF = core binding factor. *Among the 27 ELN favourable-risk patients, 21 had at least one additional high-risk feature. Percentages for the individual high-risk features are calculated out of these 21 patients.

Induction therapy consisted primarily of standard “7 + 3” chemotherapy (86.7%). As part of induction, 26.7% of patients received a FLT3 inhibitor and 36.7% received gemtuzumab ozogamicin (GO). Most patients (96.7%) achieved complete remission (CR) after a single induction cycle, with a median time to remission of 28 days (range, 15—39). Consolidation therapy most commonly included high-dose cytarabine (HiDAC)-based regimens, with the majority of patients (63.3%) receiving three cycles (range, 1–4).

### MRD status and initiation of oral azacitidine maintenance

Following induction and one consolidation cycle, molecular measurable residual disease (MRD) assessment was available in 23 NPM1 mutated patients and 2 CBF AML patients. Flow cytometry based MRD was completed in additional 3 patients, while in 2 patients MRD evaluation was not available.

The median follow-up from AML diagnosis was 26.7 months (range, 8.5–64.9). Oral azacitidine maintenance was initiated in all 30 patients after completion of intensive therapy. The median interval from AML diagnosis to oral azacitidine initiation was 191 days (range, 114—757). At the start of maintenance therapy, 14 patients (46.7%) were MRD-negative, 14 patients (46.7%) were MRD-positive, of these all but one patient, were below 2% (low positive). MRD status was unknown in 2 patients (6.7%). The median duration of oral azacitidine treatment was 12.3 months (range, 0.3—37.5).

### Safety and treatment discontinuation

Treatment discontinuation occurred in 20 patients (66.7%). The most common reason was disease progression (10 patients, 33.3%). Five patients (16.6%) completed the planned 24 months of maintenance. Treatment was discontinued due to treatment-related toxicity in four additional patients, including one with cytopenias and three with persistent low-grade but clinically burdensome adverse events, such as fatigue, abdominal pain and nausea. One patient discontinued treatment following a new diagnosis of prostate carcinoma. At the time of data cut-off, 10 patients (33.3%) remained on treatment (Table 2).

**Table 2:** Oral Azacidine treatment characteristics

Parameter	Subgroup	No. of patients (Percentage)
**Total patients treated with** Oral Azacidine	-	30
**MRD status prior to** Oral Azacidine	Negative	14 (46.4%)
	Positive	14 (46.4%)
	No MRD marker	2 (6.7%)
MRD conversion to negative (among MRD-positive patients, n = 14)	Achieved	9 (64.3%)
	Not achieved	5 (35.7%)
**Time from diagnosis to** Oral Azacidine** initiation**	Median (range), days	191 (114–757)
**Treatment discontinued**	Yes	20 (66.7%)
	No	10 (33.3%)
**Reasons for discontinuation**	Disease progression	10 (33.3%)
	Cytopenias	1 (3.3%)
	Mild continuous GI toxicity	3 (10%)
	Prostate cancer diagnosis	1 (3.3%)
	Completed ≥ 24 months	5 (16.6%)
**Time on** Oral Azacidine	Median (range), months	12.3 (0.3–37.5)

LEGEND: GI = gastrointestinal, MRD = measurable residual disease; Percentages for MRD conversion are calculated among evaluable MRD-positive patients (n = 14); all other percentages are calculated out of the total number of patients treated with Oral Azacidine (n = 30).

Adverse events were common but generally manageable. Gastrointestinal toxicity was the most frequently observed adverse event, with 16 patients (53.3%) experiencing grade 1—2 events and 2 patients (6.7%) experiencing grade 3 toxicity. Hematologic toxicity was infrequent, with a single case of grade 3 neutropenia reported. Supportive care measures, including antiemetics and dose modifications, were generally effective in mitigating treatment-related toxicity.

### Efficacy and MRD dynamics

**Fig. 1:**
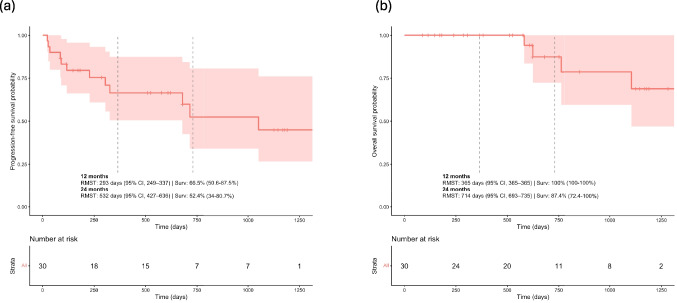
Survival outcomes. Median relapse free survival (**a**) and overall survival (**b**) for the entire cohort from the start of Onureg. For RFS, events were defined as hematologic relapse; for OS, events were defined as death from any cause

Disease relapse occurred in 13 of 30 patients (43%). 12-months restricted median relapse-free survival (RFS) was 293 days (95% CI 249—337), with estimated 1-year and 2-year RFS rates of 66.5% and 52.4%, respectively (Fig. 1a). Most relapses (11 of 13) occurred during active oral azacitidine treatment, while 2 relapses occurred after treatment discontinuation, at 83 and 359 days following cessation of oral azacitidine. With a median follow-up of 20.5 months (range, 2.9–43.9) from the initiation of oral azacitidine, 3 deaths were observed. The median overall survival (OS) was not reached, the 12-months restricted median overall survival (OS) was 365 days (95% CI 365—365), with estimated 1-year and 2-year OS rates of 100% and 87.4%, respectively (Fig. 1b).

MRD conversion, defined as loss of detectable MRD during oral azacitidine maintenance, was observed in 9 of 14 patients (64%) who were MRD-positive at baseline. During follow-up, 2 of these MRD converters experienced relapse, while 7 remained relapse-free at last follow-up. Notably, both relapses among MRD converters occurred while patients were still receiving oral azacitidine, rather than after treatment discontinuation.

We next performed OS and RFS analyses by stratifying the cohort into three groups: patients who were MRD-negative at the initiation of oral azacitidine (following induction and consolidation), MRD-positive patients who converted to MRD negativity at any time after initiation of oral azacitidine, and patients with persistent MRD positivity.

**Fig. 2:**
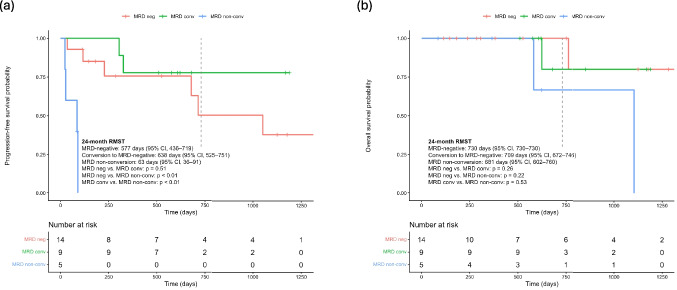
Survival outcomes baesd on MRD status. Median relapse free survival (**a**) and overall survival (**b**) for the 28 patients with evaluable MRD, stratified as patients with negative MRD at the initation of oral azacitidine (MRD negative), patients who achieved MRD negativity at any time point since the initation of oral azacitidine (conversion to negative) and patients with peristent MRD psotivity (Pos MRD). For RFS, events were defined as hematologic relapse; for OS, events were defined as death from any cause

Notably, patients who converted to MRD negativity demonstrated relapse-free survival comparable to those who were MRD-negative at initiation of maintenance therapy. Median RFS did not differ significantly between patients who were MRD-negative at treatment initiation and those who converted to MRD negativity, with a restricted 24-months RFS of 577 days (95% CI, 436—719) and 638 days (95% CI, 525—751), respectively **(**Fig. 2a, p*-value*−0.5**)**. In contrast, patients with persistent MRD positivity demonstrated a markedly shorter 24-months restricted median RFS compared with the other groups (63 days, 95% CI, 36—91) Fig. 2a). The higher relapse rate observed in the persistent MRD-positive group did not translate into a statistically significant difference in median OS, likely reflecting the ability of some patients to achieve subsequent remission with additional salvage therapies (Fig. 2b).

### Favorable with additional high-risk features

Twenty-one of the 27 (78%) favorable risk patients carried additional high-risk features (Table 1). These included age above 60 years (52%), NPM1/DNMT3A or CBF/RAS co-mutations (33%) and detectable MRD prior to initiation of oral azacitidine (67%). Eight patients had 2 high risk characterises. Relapse rate of favorable risk patients with at least one high risk feature was 43% versus 30% for patients with no high-risk feature (*p* = *0.6*).

Given the debatable prognostic significance of low level MRD, we also excluded detectable MRD from the above analysis. The relapse rate for the high-risk (HR) favorable versus the non-HR favorable were 46% and 33%, respectively (*p* = *0.4*). Median relapse free survival was 715 days (95% CI, 228 days to not reached) for the HR-favorable group compared to not-reached for the non-HR-favorable group, not reaching statistical significance.

### Post-relapse management

**Fig. 3:**
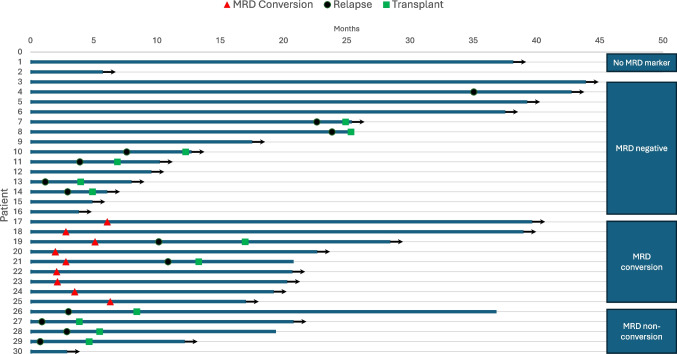
Swimmer’s plot of individual patient outcomes during oral azacitidine maintenance. Each horizontal bar represents an individual patient, with bar length indicating duration of follow-up on oral azacitidine. Colors denote MRD status and dynamics: MRD conversion, no MRD conversion, no available MRD marker, and MRD negativity prior to initiation of oral azacitidine maintenance. Black arrow signifies the patient is alive

Thirteen patients experienced relapse, including 8 with molecular relapse and 5 with overt morphologic leukemia. Five patients were treated with low-dose cytarabine or azacitidine in combination with venetoclax; all achieved a molecular response, including one patient who did not proceed to transplantation and remains in molecular remission, suggesting preserved sensitivity to HMA-based regimens despite exposure to oral azacitidine. Two patients received salvage chemotherapy with FLAG-IDA plus venetoclax. Overall, twelve patients subsequently proceeded to allogeneic stem cell transplantation, including six who did so without additional intervening therapy. Among these 12 transplanted patients, at the end of follow-up, seven were in remission and one patient was still hospitalized in the peri-transplant period. One patient relapsed after transplantation and remains alive on active treatment. Three patients died post-transplant two due to transplant-related complications and one due to relapse. Patients course is further described in Fig. 3.

## Discussion

Maintenance therapy has emerged as an increasingly important component of AML management, aimed at prolonging remission and reducing relapse risk following intensive induction and consolidation therapy [19, 20]. Several maintenance strategies have demonstrated clinical benefit, particularly in biologically defined subsets of AML, including hypomethylating agents in older or transplant-ineligible patients [21, 22], FLT3 inhibitors in FLT3-mutated disease [23, 24], and targeted agents in select molecular contexts. Collectively, these studies have shifted the post-remission paradigm from observation alone toward active intervention, providing the clinical rationale for evaluating oral azacitidine maintenance in patients with ongoing relapse risk despite achieving complete remission.

In this real-world cohort, which consisted predominantly of patients with favorable-risk AML but also included a small proportion of intermediate-risk cases, oral azacitidine maintenance was associated with encouraging MRD responses and favorable survival outcomes. These findings extend the evidence generated by the QUAZAR AML-001 trial into a population that has traditionally not been considered for maintenance therapy and for whom the optimal post-remission strategy remains ill-defined.

The QUAZAR AML-001 trial established oral azacitidine as the first maintenance therapy to significantly prolong overall survival and relapse-free survival in patients with AML in first remission who were not candidates for allogeneic transplantation [3, 4]. Long-term follow-up demonstrated that the principal benefit of oral azacitidine was prolongation of remission duration rather than complete prevention of relapse, highlighting its role as a disease-controlling rather than curative strategy [4]. Across molecular subgroups and age strata in QUAZAR AML-001, oral azacitidine maintenance consistently improved survival, but the magnitude of benefit differed by mutation and age, leaving important gaps. In patients ≥ 65 years, oral azacitidine significantly prolonged OS and RFS compared with placebo, establishing benefit in an older population at intrinsically high relapse risk. Within the NPM1-mutated subgroup (29.2% of the cohort), outcomes were heterogeneous by FLT3-ITD status: patients with NPM1-mutated/FLT3-ITD-negative AML showed a clear OS and RFS advantage with maintenance, whereas those with concurrent FLT3-ITD (~ 22% of NPM1-mutated cases) had inferior survival overall and a less pronounced maintenance benefit, consistent with the dominant adverse biology of FLT3-ITD [25]. Importantly, although ELN-favorable AML was not formally enrolled, as detailed above, a large fraction of biologically favorable NPM1-mutated (FLT3-ITD negative) patients was included, demonstrating that relapse risk in older adults persists despite favorable genetics and can be mitigated by maintenance therapy.

These findings support the concept that selected patients with otherwise favorable genetic features may nonetheless harbor biologically meaningful residual disease that is amenable to maintenance intervention. Our data align with the emerging notion of “favorable AML with additional high-risk features.” This category includes patients who meet ELN favorable-risk criteria [2] criteria but exhibit factors associated with increased relapse risk, such as low-level persistent measurable residual disease (MRD) after intensive chemotherapy [15, 26], MRD negativity accompanied by additional adverse or cooperative mutations, including *DNMT3A* or *FLT3-ITD* or clinical features such as extramedullary disease at diagnosis [9, 12, 27]. These variables are not fully incorporated into current ELN risk stratification systems yet have been consistently associated with inferior outcomes and increased relapse risk. Patients within this “high-risk favourable” category may therefore derive disproportionate benefit from maintenance strategies aimed at suppressing residual disease and delaying clonal re-expansion.

In our favorable risk cohort, 78% of the patients had at least one additional high-risk feature (age above 60 years, additional co-mutation or detectable MRD), with a relapse rate of 46%. The small number of patients, does not allow broad conclusion, particularly given the inconsistent effect of NPM1/DNMT3A co-mutations without FLT3-ITD mutation. Nonetheless, previous reports suggested a higher relapse a for these patients, especially above 60 years of age or with detectable MRD, supporting a potential role for oral azacitidine maintenance in these settings [13–15].

The relatively high rate of MRD conversion of 64% observed during oral azacitidine maintenance, represents a central finding of this study and is significantly higher than previous reports of “spontaneous” MRD conversion rate of 30% [28], supporting the biological plausibility that hypomethylating agents can deepen molecular responses and potentially alter disease trajectory. Importantly, patients who achieved MRD conversion had relapse-free survival comparable to those who were MRD-negative at baseline, suggesting that conversion to MRD negativity may mitigate the adverse prognostic impact of baseline MRD positivity. In contrast, patients with persistent MRD positivity experienced markedly inferior RFS, underscoring the prognostic relevance of sustained MRD despite maintenance therapy.

Although MRD assessment was largely standardized using molecular assays, a small subset of patients was evaluated using flow cytometry, which may introduce inter-laboratory variability. However, this is unlikely to have impacted the MRD conversion findings, as conversion events were observed exclusively in molecularly assessed patients.

Notably, the higher relapse rate observed among persistently MRD-positive patients did not translate into a statistically significant difference in overall survival. This dissociation between RFS and OS may reflect the effect of subsequent therapies, including additional lines of treatment and allogeneic transplantation, but should also be interpreted in the context of the relatively short follow-up duration, limited cohort size, and small number of events.

Indeed, although survival outcomes were favorable overall, with median OS not reached even among patients with baseline MRD positivity, the small number of patients in each MRD-defined subgroup precludes definitive conclusions regarding the survival impact of MRD conversion. Nevertheless, these findings are concordant with real-world data and extended analyses of the QUAZAR AML-001 study [4, 25, 29], which demonstrated durable remission prolongation with oral azacitidine maintenance, and further emphasize the need for refined biomarkers to identify patients most likely to derive sustained benefit from maintenance strategies.

A potential concern with prolonged exposure to hypomethylating agents is the development of resistance to azacitidine, which could compromise subsequent therapy. Proposed mechanisms include altered drug uptake and metabolism, epigenetic reprogramming, clonal evolution, and selection of pre-existing resistant subclones. Notably, in our cohort, responses to venetoclax-based salvage regimens following relapse on oral azacitidine were preserved, arguing against clinically meaningful cross-resistance to azacitidine. Although patient numbers are limited, these findings are reassuring and confirm previous reports [29], suggesting that oral azacitidine maintenance does not preclude effective subsequent treatment with hypomethylating agent–based combinations.

Toxicity in our cohort was manageable and consistent with the known safety profile of oral azacitidine. Gastrointestinal adverse events were the most common and were generally low grade, with infrequent need for dose modification. Hematologic toxicity was uncommon. These findings mirror those reported in QUAZAR AML-001 and multiple real-world series, supporting the feasibility of prolonged maintenance therapy in routine clinical practice [4, 5].

This study has several important limitations. First, the relatively small sample size limits statistical power and precludes definitive subgroup analyses. Second, the retrospective design introduces potential selection bias and limits control over confounding variables. Third, the absence of a control group prevents direct assessment of treatment efficacy compared to observation or alternative strategies. As such, our findings should be interpreted as hypothesis-generating rather than practice-changing.

In summary, this real-world cohort of patients with predominantly favourable risk AML demonstrates that oral azacitidine maintenance is associated with meaningful MRD responses and favourable survival outcomes, extending the applicability of maintenance therapy beyond traditionally high-risk populations. Importantly, MRD dynamics, particularly conversion to MRD negativity, emerged as a key determinant of relapse risk. The concept of favourable AML with additional high-risk features may help refine patient selection by identifying individuals who, despite favourable baseline genetics, remain at clinically significant risk of relapse. Our findings are not practice-changing but support the concept of MRD-guided maintenance strategies. Future trials, ideally prospective and incorporating MRD as a stratification factor and endpoint, are needed to define the role of oral azacitidine in patients with “high-risk favorable” AML.

## Data Availability

The data that support the findings of this study are available from the corresponding author upon reasonable request.
